# A cross-sectional study on the association between varied social support modalities and glycemic levels amongst diabetic patients residing in Machakos County, Kenya

**DOI:** 10.11604/pamj.2023.45.99.39472

**Published:** 2023-06-22

**Authors:** Jackline Njeri Kiarie, Susan Njoki Mambo, George Kimathi Kamundi

**Affiliations:** 1 School of Public Health, Department of Environmental Health and Disease Control, Jomo Kenyatta University of Agriculture and Technology, Nairobi, Kenya,; 2 School of Public Health Jomo Kenyatta University of Agriculture and Technology, Nairobi, Kenya,; 3 Amref International University, Nairobi, Kenya

**Keywords:** Diabetes type 2, social support, glycemic control, Africa

## Abstract

**Introduction:**

diabetes is a chronic disease that occurs either when the pancreas does not produce enough insulin or when the body cannot effectively use the insulin it produces. While there's increasing evidence that social support from caregivers improves health outcomes in chronic illness management, the potential associations of the different types of social support and glycemic control among Type II diabetes clients have largely been ignored in Kenya. This cross-sectional study sought to establish the association between tangible, emotional, and informational social support and glycemic levels among clients diagnosed with Type II diabetes in Machakos County, Kenya.

**Methods:**

semi-structured interviews were conducted with 726 randomly selected Type II diabetes clients enrolled in diabetes care and treatment programs in government-owned public health facilities in Masinga and Matungulu sub-counties, Machakos, Kenya. Descriptive statistics and multinomial logistic regression were conducted to elucidate any associations.

**Results:**

seventy-three percent (73%) of the respondents were female, and 27% were male, with the majority (77.9%) being above 50 years and having lived with diabetes for over 3 years (61.5%). Opportunities for social support existed, with 62% of the respondents living with more than 2 persons above 18 in their households. From the Random Blood Glucose test analysis, 38.9% of the clients had high glycemic levels, partly because the majority (66.9%) of the respondents did not practice good diabetes self-management practices at the time of the study. While all three types of social support were reported as provided, only 30.6% reported receiving adequate social support. An association was found between social support and glycemic levels with respondents receiving adequate informational- P<0.05, OR 1.92, emotional -P<0.05, OR 3.7, and tangible support -P<0.05, OR 4.1 more likely to have better glycemic control than those with inadequate support.

**Conclusion:**

clients receiving adequate informational, emotional, and tangible social support were 2, 4, and 4 times, respectively, likely to have better glycemic control than those with inadequate support. Of the three types of social support, tangible support was most needed. Ultimately, a greater understanding of these interactions through longitudinal studies is required to identify solutions and optimize glycaemic control for diabetes clients in Kenya and beyond.

## Introduction

Background/rationale: Diabetes is a chronic disease that occurs either when the pancreas does not produce enough insulin or when the body cannot effectively use its insulin, a hormone that regulates blood sugar. WHO diagnostic criteria for diabetes defines fasting plasma glucose as ≥ 7.0mmol/l (126mg/dl) or 2-hour plasma glucose ≥ 11.1mmol/l (200mg/dl) [1]. Type II diabetes is amongst the most prominent NCDs in sub-Saharan Africa (SSA), with an estimated 15.5 (9.8-27.8) million adults living with Type II diabetes as of 2017, translating to a regional prevalence of ~?6% and associated healthcare costs of USD 3.3 billion [2]. In Kenya, for example, the Type II diabetes prevalence is estimated at 4.2% of the general population, with 2.2-2.7% reported in rural areas and 10.7-12.2% in urban areas accounting for more than 50% of total hospital admissions and over 55% of hospital deaths [3,4]. Management of Type II diabetes involves self-management practices to improve glycemic control. They involve adherence to a complex regimen of pharmacological management practices, such as using medicines (oral hypoglycemics or insulin therapy), and non-pharmacological management practices, such as a healthy diet, exercises, glucose self-monitoring, and foot care [5,6]. There is a need to address these barriers to self-management to delay or prevent the development of diabetic complications [7]. The self-management activities can be complex, making it difficult for most diabetic patients to meet self-management goals [8].

Social support is a multidimensional concept that refers to the psychological sense of belonging, acceptance, and assistance due to support from family, friends, and social networks. Regardless of the source, social support is often measured in three dimensions: tangible, emotional, and informational social support [9]. Tangible support refers to visible and sometimes material assistance from others to complete tasks and manage problems, such as financial assistance, blood sugar monitoring, meal preparation, hospital accompaniment, etc. The second type of support is emotional support which enhances the experience of being respected, supported and understood. It provides comfort and affection like empathy, trust-building, affirmations, and promotion of self-esteem to encourage and build confidence to reinforce self-care and skills/abilities. The third type of support is informational support which facilitates access to information in the form of advice, recommendations, facts, and information or access to social networks that provide a sense of information and peer encouragement in Type II diabetes self-management [10-13].

There's an urgent need to adapt health systems to improve Type II diabetes self-management at the most basic level, which is at the environment in which persons living with Type II diabetes and their caregivers are based [14-16]. Informal supporters such as primary caregivers, who include spouses or partners of people living with Diabetes (SPWD) who often live in the same household with diabetic patients, are better positioned to provide social support to influence a patient's day-to-day diabetes self-management practices [17,18]. Yet, investigations of their influence on daily diabetic patient health behaviors, especially in Africa, are limited. Unfortunately, most evidence, including a systematic review of studies of 'international scope' have focused on America, Asia, and Europe, with very little evidence from the African region. This limits the evidence base on how best to structure social support in improving adherence to self-management for diabetes clients in Low and Middle-Income Countries (LMICs) such as Kenya [3,15,19]. Recommendations have also been made on the need to disaggregate the components of social support associated with improved glycemic control in future research [20]. Therefore, this study sought to extend the evidence base to improve understanding of which aspects of social support are associated with glycemic control capabilities amongst clients with Type II diabetes in Machakos County, Kenya.

**Broad objective:** to establish the association between different types of social support and glycemic control amongst Type II diabetes patients in Machakos County. Specific objectives: 1) to determine social demographic factors associated with glycemic levels amongst Type II diabetes clients in Machakos County; 2) to determine the types of social support provided to Type II diabetes clients in Machakos County; 3) to establish the relationship between different types of social support and glycemic control amongst Type II diabetes clients in Machakos County. Study null hypothesis - (No): Social support is not associated with glycemic control amongst patients living with Type II diabetes in Machakos County. Study alternate hypothesis (H1): Social support is associated with glycemic control amongst patients with Type II diabetes in Machakos County.

## Methods

**Study design:** the study employed a cross-sectional design that adopted quantitative data collection and an alysis approaches.

**Study area:** the study was conducted in Machakos County, one of the 47 counties in Kenya, with a population of 1,421,932 people (KNBS, 2019). The County has 416 health facilities, with 6% offering health services to reverse the rising burden of non-communicable conditions (Kenya Master Health Facility List, 2020). The County has been reported amongst the top counties reporting elevated glucose levels, with DHIS 2 data indicating over 15,000 persons with diabetes. The study was conducted in Matungulu and Masinga sub-counties, rural sub-counties in Kenya, to promote the generalization of knowledge acquired to 68.9% of the Kenyan population living in rural areas and the larger African region.

**Study participants:** the study purposefully identified clients diagnosed with diabetes and enrolled in the Type II diabetes care and treatment program in government-owned public health facilities in the Masinga and Matungulu sub-counties who were between 18 and 65 years of age, who could read and write and lived with a person above 18 years who could serve as a primary caregiver. The primary caregiver included persons such as a family member, relative, etc., who lived in the same household with the diabetic patient. Identifying study respondents from the health facility had the advantage of a physician-confirmed diagnosis of Type II diabetes clients and provided a channel to access the patient. The relationship between social support and glycemic levels was assessed from the client's perspective.

**Figure 1:**
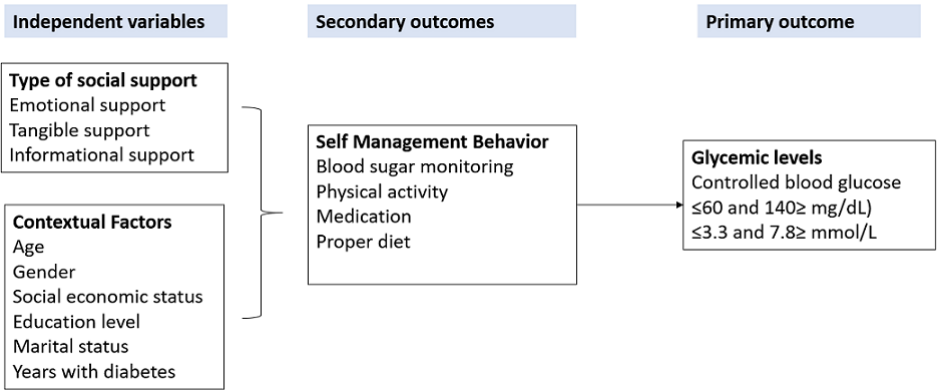
study conceptual framework

**Study variables:** the independent variables in the study included the different types of social support, such as emotional support, informational support, and tangible support. The study also captured contextual factors such as patient demographic characteristics as independent variables. The dependent variable was glycemic control, measured using a random blood sugar test by a health worker during the monthly diabetes clinic visit. The conceptual framework in Figure 1 summarises the relationship between the study variables;

**Data sources:** health facilities providing Type II diabetes health services were identified. The list of Type II diabetes clients enrolled in the diabetic clinics in these health facilities formed the sampling frame for each sub-county. Initial engagement with the health workforce in the health facilities revealed a total caseload of 2,171 clients with complete health records enrolled in these health facilities, 74% female (1596) and 26% male (575). The mean age of the enrolled clients was 57 years, with a minimum of 18 and a maximum of 90 years. Data collection was through a semi-structured questionnaire on the Open Data Kit (ODK) that captured the demographic characteristics of the study group, efficacy in Type II diabetes self-management, and extent of social support being received by the respondent. Twenty research assistants with college-level education and who own Android-supported SMART mobile devices from the two localities were identified, recruited, and trained on the scope of the study and data collection procedures. Random Blood Glucose (RBG) tests on capillary blood were performed by the nurse or clinical officer using a standard glucometer at the health facility where the clients accessed their diabetes care during the data collection days, which coincided with the NCD clinic days. To ensure alignment with current standard practice in most rural public health facilities in Kenya, the RBG test was used in the study to monitor blood sugar levels amongst diabetes clients. Ethical clearance was obtained from the Baraton University Ethics and Research Committee and other County Health Research Board approvals.

**Bias:** while consent was sought in recruiting study respondents, those who refused to participate were automatically excluded, which in itself may have introduced biases given that some of their reasons for refusing to participate may have been influenced by the quality of relationships the patients living with diabetes had with their primary caregivers, a critical factor in the study.

**Sample size determination:** Cochran's formula for determining the appropriate sample size was utilized to determine the appropriate sample size for the research [21], n is the sample size, z is the selected critical value or desired confidence level, p is the estimated proportion of an attribute that is present in the population, q p = -1, and e is the desired level of precision, which gave a sample size of 384.


$$n=\frac{{}^{Z}\left(\alpha /2\right)xpx\left(1-p\right)}{e^{2}}$$

$$x=\frac{1.96^{2}x0.9x\left(1-0.9\right)}{0.0343^{2}}=384$$

A design effect of 2 was applied to accommodate the samples from the two sub-counties, making a minimum sample size of 768 with additional respondents identified to accommodate loss to follow-up. Study respondent allocation across the two sub-counties was made proportionately to size, with 33% being identified from the Matungulu sub-county and 67% from the Masinga sub-county. During sampling, the clients with complete records were extracted from the client register, coded, and loaded on a 'Research Randomizer' digital randomization software for sampling. A sampling interval of 'three' was applied to meet the targeted sample size from the complete sampling frame. The identified respondents were invited to consent to the study. For confidentiality purposes, the patient records were extracted from the hospital records without personal identifiers like names.

**Self-management behavior:** self-management amongst clients living with diabetes refers to the active participation of patients in their care through education on diabetes and its management, monitoring blood glucose levels, medication management, healthy eating, physical activity, and coping with the psychosocial challenges associated with living with diabetes. These practices were investigated during the study through 8 positive and 7 negative Likert scale questions. To determine overall self-management behavior, the responses were coded as 1- Strongly agree, 2- Agree, 3-Neutral, and 4- Not at all. For positive statements, a score of 1-2 was categorized as good self-management and 3-4 as poor self-management. A score of 4 for a negative statement was categorized as good self-management and 1-3 as poor self-management. The 15 responses were coded as 0 -Poor self-management and 1 Good self-management.

**Social support needs of clients living with diabetes:** the extent of social support received was assessed using three constructs: emotional, informational, and tangible social support. The extent of emotional support received was measured using four indicators. These include the need for someone to count on to listen when in need to talk, someone to share most private worries and fears with, someone who understands the client's problems, and someone to confide in or talk to about personal issues. The extent of informational support received was assessed using four indicators. These included the need to have someone to give the participant's information and to help them understand a situation, the need for someone to turn to for suggestions about how to deal with a personal problem, some to give the participant good advice about a crisis, and someone whose advice the participant wants. The last construct of social support assessed was the extent of tangible support received. It was assessed based on five indicators which included the need for someone to help if confined to bed, someone to take the patient to the doctor when the need arises, someone to prepare meals when the participant is unable to do it themselves, and someone to help with daily chores when the participant is sick, and someone to support with physical activities.

**Data processing and analysis:** a total of 726 eligible individuals were successfully interviewed amongst the targeted sample of 768 persons living with Type II diabetes, yielding a response rate of 95%. Data entry was performed using KoboToolbox then exported to Statistical Package for Social Sciences (SPSS) version 19.1 (Predictive Analytics Software) for data cleaning to enhance data quality. Data cleaning through crosschecking for errors was conducted in readiness for processing and analysis. Missing values were less than 5% for most items. The analysis included descriptive statistics of frequencies, and percentages, to summarise key variables on socio-demographic characteristics, self-management practices, and social support needs. To determine overall self-management behavior, the responses were coded as 1- Strongly agree, 2- Agree, 3-Neutral, and 4- Not at all. For positive statements, a score of 1-2 was categorized as good self-management and 3-4 as poor self-management. A score of 4 for a negative statement was categorized as good self-management and 1-3 as poor self-management. The 15 responses were coded as 0 -Poor self-management and 1 Good self-management. A composite variable was generated by adding the 15 responses, and a score of 12 and above, or 80% and above, was considered good self-management. Similarly, a combined composite variable of emotional, informational, and tangible support to determine the overall level of social support provided was developed. A total score of 3 was categorized as adequate overall support, while 0-2 was categorized as inadequate overall support. Inferential statistical analysis that included logistic regression analysis at a 95% confidence interval of participants' glycemic sugar levels and socio-demographic characteristics and a multinomial logistic regression analysis to determine the extent of association between informational, emotional, and tangible support with glycemic levels were also conducted. The results are presented in frequency distribution tables, charts, and graphs.

## Results

**Socio-demographic information:** of the 726 respondents, 324 persons (45%) of the sample were drawn from Masinga Sub-county, and the remaining 402 persons (55%) were from the Matungulu sub-county. Grouped by gender, analysis shows that 530 persons representing 73% of the sample, were female, while 27% were male. An analysis of the level of education showed that 88.8% of the clients had some form of formal schooling. Forty-six point six percent of the clients were also either employed or self-employed, providing self-sustenance to meet the self-management requirements. A critical factor in the study was marital status which somehow influences the ability to get a primary caregiver in the same household. As depicted in Table 1, 73% were married, most of whom were older persons above 50 (78%) and had lived with diabetes for over two years (71%), making them suitable respondents for this study given their experience living with diabetes. For each household, the population of people above 18 years ranged between 0-11. A higher proportion of participants reported living with at least two people (33.6%) and at least three (28.4%) aged 18 and above, respectively.

**Table 1:** social demographic characteristics of study respondents (n=726)

Variable	Category	Overall n (%)	Masinga	Matungulu
Sex	Male	196(27)	91(28.1)	105(26.1)
	Female	530(73)	233(71.9)	297(73.9)
Highest level of education	No formal schooling	81(11.2)	42(13.0)	39(9.7)
	Primary	337(46.4)	154(47.5)	183(45.5)
	Secondary	218(30)	92(28.4)	126(31.3)
	College	90(12.4)	36(11.1)	54(13.4)
Employment status	Employed	82(11.3)	39(12.0)	43(10.7)
	Self-employed	256(35.3)	104(32.1)	152(37.8)
	Non-paid/volunteer	238(32.8)	109(33.6)	129(32.1)
	Homemaker (housewife/househusband)	92(12.6)	41(12.7)	51(12.6)
	Unemployed (able to work)	5(0.7)	2(0.6)	3(0.7)
	Unemployed (unable to work)	53(7.3)	29(9.0)	24(6.0)
Marital status	Single/never married	26(3.6)	12(3.7)	14(3.5)
	Married	530(73)	222(68.5)	308(76.6)
	Divorced/separated	33(4.5)	15(4.6)	18(4.5)
	Widowed	137(18.9)	75(23.1	62(15.4)
Age of participants	18-29	5(0.7)	2(0.6)	3(0.7)
	30-39	10(1.4)	6(1.9)	4(1.0)
	40-49	145(20)	60(18.6)	85(21.1)
	50-59	135(32.5)	107(33.2)	128(31.8)
	60-69	192(26.5)	91(28.3)	101(25.1)
	70-79	123(17)	53(16.5)	70(17.4)
	80-89	14(1.9)	3(0.9)	11(2.7)
Years living with diabetes	Less than six months	43(6.1)	19(6.1)	24(6.0)
	One year	164(23.1)	76(24.3)	88(22.2)
	Two years	66(9.3)	28(8.9)	38(9.6)
	3 or more years	437(61.5)	190(60.7)	247(62.2)

**Self-management behavior:** Table 2 summarizes the extent of diabetes self-management practices among the study respondents. Only a minority (33.1%) of the participants reported practicing good self-management; When probed further on the reason for poor self-management practices, a majority (67.9%) of the respondents blamed a lack of a blood sugar measuring machine, a lack of information on the importance of physical exercise during diabetes self-management (56.5%), inability to afford a balanced diet (50%) and a lack of drugs at the health facility (42.1%) for the poor self-management practice.

**Table 2:** self-management practices of study respondents in Machakos County

Reported self-care practices	Proportion of participants			
	Strongly agree	Agree	Neutral	Not at all
I check my blood sugar levels with care and attention	52.9	21.5	15.7	9.9
The food I choose to eat makes it easy to achieve optimal blood sugar levels	35.5	38.8	21.5	4.1
I keep all doctors' appointments recommended for my diabetes	62.8	21.5	10.7	5
I take my diabetes medication (e.g., insulin, tablets) as prescribed	67.8	19	8.3	5
Occasionally I eat lots of sweets and other foods rich in carbohydrates	10.7	5.8	16.5	66.9
I record my blood sugar levels regularly to monitor my blood sugar levels	21.5	18.2	37.2	23.1
I tend to avoid diabetes-related hospital visits	10.7	5.8	12.4	71.1
I do regular physical activity to achieve optimal blood sugar levels	34.7	35.5	22.3	7.4
I strictly follow the dietary recommendations given by my doctor or diabetes specialist	23.1	28.9	38.8	9.1
I do not check my blood sugar levels frequently enough as would be required for achieving good blood glucose control	17.4	26.4	22.3	33.9
I avoid physical activity although it would improve my diabetes	6.6	3.3	26.4	63.6
I tend to forget to take of skip my diabetes medication (e.g., insulin, tablets)	8.3	5	13.2	73.6
Regarding my diabetes care, I should visit the health facility whenever I suspect a diabetes-related complication, such as wounds that do not heal	66.9	17.4	9.1	6.6
I tend to skip planned physical activity	5.8	5	17.4	71.9
My diabetes self-care is poor	11.6	6.6	24	57.9

**Extent of social support for clients living with diabetes:** the indicators of extent of emotional support provided were measured using a Likert scale as depicted in Table 3 in which a score of 0 showed that the participant doesn't receive emotional support, 1 is a little emotional support some of the time, 2 is some of the time, 3 is most of the time, and 4 is all the time. A composite variable constituting the 4 aspects of emotional support was generated to measure overall emotional support. A score of 3 was used as a cut-off point to categorize inadequate emotional support, with 1-3 coded as 0 and adequate emotional support to be 4-5 coded as 1. A score of at least 3 out of 4 was reported as adequate emotional support. The results showed that overall, 58.9% of the respondents felt they were receiving adequate emotional support from their primary caregivers. The indicators of the extent of informational support were measured using a Likert scale as depicted in Table 3 in which a score of 0 showed that the participant needs informational support none of the time, 1 is a little of the time, 2 is some of the time, 3 is most of the time, and 4 is all the time. A composite variable constituting the 4 aspects of informational support was generated to measure overall informational support. A score of 3 was used as a cut-off point to categorize inadequate informational support, with 1-3 coded as 0 and adequate informational support to be 4-5 coded as 1. A score of at least 3 out of 4 was reported as adequate informational support. From the analysis, 57% of the respondents reported receiving adequate informational support.

**Table 3:** extent of social support received by clients living with diabetes in Machakos County

Emotional support	None of the time	A little of the time	Some of the time	Most of the time	All the time
Someone you can count on to listen to you when you need to talk	17.9	6	14.5	31.6	29.9
Someone to share your most private worries and fears with	17.8	5.9	16.1	29.7	30.5
Someone who understands your problems	18	7.2	9.9	31.5	33.3
Someone to confide in or talk to about yourself or your problems	16.8	7.1	14.2	26.5	35.4
**Informational support**	**None of the time**	**A little of the time**	**Some of the time**	**Most of the time**	**All the time**
Someone to give you information to help you understand a situation	20	5.8	14.2	26.7	33.3
Someone to turn to for suggestions about how to deal with a personal problem	18.5	9.2	14.3	25.2	32.8
Someone to give you good advice about a crisis	20	5.8	13.3	32.5	28.3
Someone whose advice you really want	20.7	6.9	12.1	26.7	33.6
**Tangible support**	**None of the time**	**A little of the time**	**Some of the time**	**Most of the time**	**All the time**
Someone to help you if you were confined to	13.3	7.5	10.8	27.5	40.8
Someone to take you to the doctor if you	14.4	5.1	16.1	28	36.4
Someone to prepare your meals if you were	12.8	7.7	6.8	27.4	45.3
Someone to help with daily chores if you were	12.5	7.5	7.5	33.3	39.2
Someone to do something physical activity with	13.9	8.9	16.8	28.7	31.7

**Figure 2:**
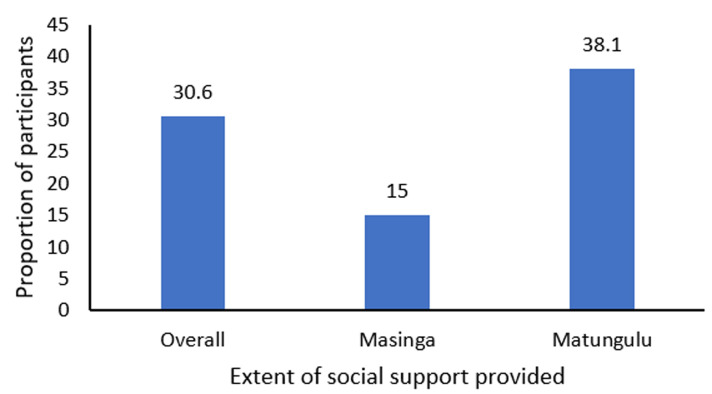
extent of social support provided

The indicators of the extent of tangible support received were measured using a Likert scale as depicted in Table 3, in which a score of 0 showed that the participant needs tangible support none of the time, 1 is a little of the time, 2 is some of the time, 3 is most of the time, and 4 is all the time. A composite variable constituting the 5 aspects was generated to measure the overall tangible support received. A score of 3 was used as a cut-off point to categorize inadequate tangible support, with 1-3 coded as 0 and adequate tangible support coded as 4-5 coded as 1. A total score of 5 was reported as adequate tangible support. From the analysis, 57% of the respondents reported receiving adequate tangible support. The analysis further developed a combined composite variable of emotional, informational, and tangible support to determine the overall level of social support provided. A total score of 3 was categorized as adequate overall support, while 0-2 was categorized as inadequate overall support. From the analysis, it was clear that overall, only 30.6% of the study respondents were receiving adequate social support at the time of the study, as depicted in Figure 2.

**Figure 3:**
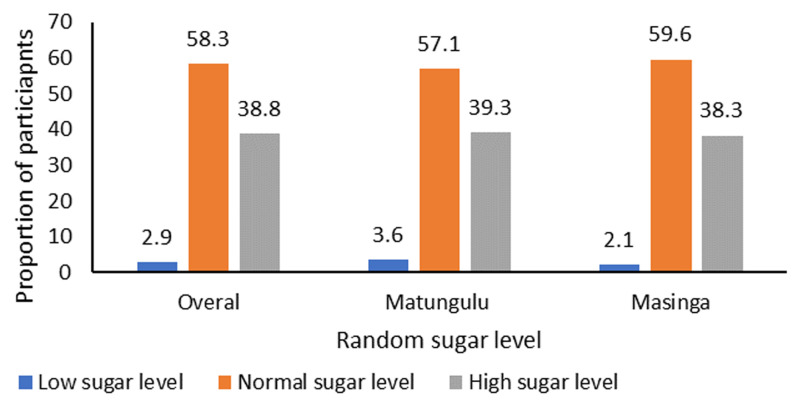
glycemic levels among study respondents

The relationship between socio-demographic characteristics, social support, and glycemic control was determined using logistic regression analysis. The blood sugar level is the crucial target indicator for glycemic control among people with diabetes. There are various methods of measuring blood sugar levels. The study conducted random blood glucose (RBG) tests using a standard glucometer to measure the blood sugar levels of diabetic clients during clinic days. Random blood glucose test is a medical diagnostic test used to measure the glucose level in the blood at any given time without regard to when the person last ate, which is the most common approach used in most rural public health facilities in Kenya. In the study, a health worker (nurse or clinical officer) conducted the random blood sugar test during the non-communicable disease (NCD) clinic date. Blood glucose test scores above 11 mmol/L were classified as high sugar levels. During analysis, the sugar levels between 1-4 mmol/L were categorized as low, 5-11 mmol/L as normal, and 11.1 mmol/L and above as high. From the analysis, 38.9% of the study respondents had high sugar levels as depicted in Figure 3. Inferential statistics were further conducted to elucidate any association of the blood sugar levels with the socio-demographic characteristics and social support received. Table 4 shows the logistic regression analysis at a 95% confidence interval of participants' glycemic sugar levels and socio-demographic characteristics. From the findings, none of the socio-demographic characteristics was associated with the sugar levels among the study participants.

**Table 4:** logistic regression analysis of socio-demographic characteristics and social support

Variables	B	df	Sig.	Exp(B)	95% Confidence Interval for Exp(B)	
					Lower bound	Upper bound
Male	0.022	1	0.911	1.022	0.697	1.501
Female						
No schooling	-0.327	1	0.393	0.721	0.341	1.527
Primary	-0.096	1	0.738	0.908	0.517	1.596
Secondary	-0.072	1	0.807	0.93	0.521	1.661
College						
Employed	0.412	1	0.301	1.511	0.692	3.295
Self-employed	0.066	1	0.845	1.068	0.551	2.071
None paid	0.135	1	0.695	1.144	0.584	2.242
Student	-16.922	1	0.996	4.478	0	.c
Homemaker	0.283	1	0.486	1.327	0.598	2.945
Unemployed-able to work	-1.355	1	0.261	0.258	0.024	2.734
Unemployed-unable to work						.
Single	0.349	1	0.492	1.418	0.523	3.843
Married	-0.098	1	0.661	0.907	0.585	1.404
Divorced	-0.017	1	0.969	0.983	0.428	2.261
Widow						
Age 18-29	0.575	1	0.655	1.778	0.143	22.138
Age 30-39	-0.972	1	0.326	0.378	0.054	2.631
Age 40-49	-0.387	1	0.552	0.679	0.191	2.426
Age 50-59	-0.304	1	0.633	0.738	0.211	2.575
Age 60-69	-0.14	1	0.828	0.871	0.247	3.065
Age 70-70	-0.514	1	0.429	0.598	0.167	2.141
Age 80-89						

On the other hand, a multinomial logistic regression analysis revealed that informational, emotional, and tangible support were significantly associated with glycemic levels. Participants receiving adequate informational P<0.05, OR 1.92, emotional -P<0.05, OR 3.7, and tangible support -P<0.05, OR 4.1 were more likely to have normal sugar than those with inadequate support, as shown in Table 5. Participants receiving adequate informational, emotional, and tangible support were 2, 4, and 4 times more likely to report normal sugar levels, respectively, than those who didn't.

**Table 5:** multinomial logistic regression analysis of different types of social support and glycemic levels

Blood sugar level	Types of support	B	df	Sig.	Exp(B)	95% Confidence Interval for Exp(B)	
						Lower Bound	Upper Bound
Low sugar	Overall informational support	-0.748	1	0.326	0.473	0.106	2.105
	Overall emotional support	-20.543	1	0.998	1.201	0	
	Tangible support	21.039	1	.	1371326972	1371326972	1371326972
	Overall self-care	0.441	1	1	1.555	0	
Normal sugar	Informational support	0.653	1	0.024	1.921	1.088	3.389
	Emotional support	1.317	1	0	3.733	1.935	7.2
	Tangible support	1.411	1	0	4.099	2.369	7.09
	Overall self-care	0.102	1	0.755	1.107	0.585	2.096

The reference category is high sugar level

## Discussion

This study sought to determine the association between the different types of social support and glycemic control amongst clients living with diabetes in Machakos County, a peri-urban environment in Kenya. As recommended in previous studies, this study delved deeper into the different types of social support: tangible, emotional, and informational social support [22]. Blood sugar monitoring using a random glucose test was conducted on the day of the study to determine the glycemic levels amongst the respondents. A Random Blood Glucose (RBG) test involves taking a blood sample randomly, no matter when the client ate last. A blood sugar level of 200 milligrams per deciliter (mg/dL) - 11.1 millimoles per liter (mmol/L) - or higher is considered high [23]. In this study, 38.9% of the respondents had RBG scores of 11.1 mmol/L and higher, suggesting poor glycemic control. Suboptimal glycemic control is pervasive amongst patients with type-2 diabetes in SSA, as seen in countries like Ethiopia, where 70.1% of respondents in a similar study were found to have high glycemic levels [24] and 70% across a systematic review of 74 studies involving 21,133 participants [25], ultimately posing a public health challenge in the region. The difference in glycemic levels noted between these studies could be partly because of the different blood sugar tests used in measuring blood sugar levels; the random blood sugar test, which only classifies glycemic RBG scores of 11.1 mmol/L as high blood sugar as opposed to the fasting blood sugar test which cuts off at 7.8 mmol/L. The study was also conducted when the country was experiencing a drought season [26]. Regardless of whether it is a high or low-quality diet, food insecurity is significantly associated with elevated blood sugar levels, recommending increased resourcing for people with food insecurity for optimal diabetes management [27].

Diabetes self-management is strongly associated with glycemic control in Type 2 diabetes populations [28]. Poor self-management practices were noted in the study, where only a minority (33.1%) of the participants reported practicing good self-management. This behavior was previously observed in a systematic review and meta-analysis conducted in Ethiopia, which revealed that more than half of diabetic patients in the country had poor diabetes self-care practices [29]. The primary reason for poor self-management behavior was a lack of a glucometer (67%), a common occurrence in Africa where the presence of a glucometer at home has been shown to have a positive association with reported good medication adherence amongst adult diabetic patients [30]. Given that the reasons for poor blood sugar monitoring are easily modifiable, government-subsidized initiatives to promote the use of glucometers through subsidies and awareness creation can improve blood sugar monitoring by clients living with Type II diabetes [31]. In most of these studies, the longer a client had lived with diabetes, the higher the association with worse glycemic control. In this study, 61.5% of the clients had lived with diabetes for more than three years, which could partly explain the complacency in observing proper self-management practices - only 33.1% observed good diabetes self-management practices. Several theories, such as the self-regulation model of illness behavior or the health belief model (HBM), could be used to explain this [32]. The theories suggest that as individuals' beliefs and attitudes about their illness and self-management practices change over time, their likelihood of engaging in effective self-management practices may also change [33]. Other reasons given for the poor self-management practices were a lack of information on the importance of physical exercise during diabetes self-management (56.5%), inability to afford a balanced diet (50%), and a lack of drugs at the health facility (42.1%) for the poor self-management practice. While self-management has been reported as a practical approach to improved glycemic control in diabetics, the means to sustain it has been lacking [34]. The social cognitive theory explains that self-efficacy - the ability of a person living with a chronic illness to express confidence in their ability to undertake specific self-management behaviors - is the primary influence on successful self-management [35]. The theory further recognizes that self-efficacy doesn't happen in a vacuum but that environmental factors such as support from formal health systems, health care workers, and informal social network members, including the physical environment, significantly, directly, or indirectly influence self-management behavior [36].

This study delved deeper into the three forms of social support and their association with glycemic control. This is because the lack of social support in diabetes management programs has been associated with worse health status, diabetes complications, and greater urgent healthcare use, which called for the continued investigation of the social support needs of clients with diabetes [37]. While 58.9% of the respondents felt they received adequate emotional support, 57% adequate informational support, and 57% adequate tangible support from their caregivers, put all together, only 30.6% of the study respondents were receiving adequate social support from their primary caregivers at the time of the study. This is an unfortunate situation given that the better the quality of social support provided, the better the adherence to diabetes self-care behaviors, ultimately leading to improved blood sugar control and diabetes complications [38]. In Ethiopia, for example, social support was reported to improve self-management adherence and glycemic level control [9].

This study's multinomial logistic regression analysis revealed that informational, emotional, and tangible support were significantly associated with sugar levels. Participants receiving adequate informational- P<0.05, OR 1.92, emotional -P<0.05, OR 3.7, and tangible support -P<0.05, OR 4.1 were more likely to have normal glycemic levels than those with inadequate support. This level of analysis was necessary because previous studies have primarily focused on the general association of social support and glycemic control, ignoring the potential association between specific types of social support and glycemic control [39]. From the findings, participants receiving adequate tangible, emotional, and informational support were 4, 4, and 2 times more likely to report normal sugar levels, respectively, than those who didn't. These results also depicted that people with diabetes reported a higher need for tangible support than other types of social support. Tangible support often included material assistance from others to complete tasks and manage problems, such as financial assistance related to self-management in Type II diabetes care, blood sugar monitoring, meal preparation, hospital accompaniment, etc. This finding contradicts some studies that found informational support (49.26%) is the most important type of support, followed by tangible support (39.24%) and emotional support [40]. This discrepancy in findings can be partly attributed to the high poverty levels in rural counties in Kenya, as depicted in the 2020 Economic Survey Report in Kenya, where rural counties such as the two sub-counties covered in the study were found to have high poverty rates as compared to the urban and peri-urban counties [41]. Poverty levels imply that most community members will not focus on needs higher up in Maslow's hierarchy of needs, such as emotional support, until the most basic fundamental needs, such as food through tangible support, are met [42]. That said, these findings emphasized the need to recognize different types of social support as key to diabetic self-management programs and hence structure social support involving health care providers, patients, and their social support network for diabetes self-management to improve health outcomes within this population cohort. Overall, these findings are aligned with the Optimal Matching Theory (OMT), which hypothesizes that the effects of social support are enhanced when its provision is matched with the need for support.

**Study limitations:** the study was also conducted in one County covered by one community in Kenya, implying that cultural biases may have come into play. Also, due to the cross-sectional nature of the study, causal relationships could not be determined.

## Conclusion

This study revealed that 38.9% of clients with Type II diabetes in Machakos County, Kenya, had high glycemic levels at the time of the study. Participants receiving adequate informational- P<0.05, OR 1.92, emotional -P<0.05, OR 3.7, and tangible support -P<0.05, OR 4.1 were more likely to have normal glycemic levels than those with inadequate support. While all three types of social support are received by clients living with diabetes, only 30.6% reported receiving adequate social support, with tangible support being the most needed. This could have contributed to only a minority (33.1%) of the participants practicing good diabetes self-management practices. These findings present an opportunity to address the factors identified to improve glycaemic control. Community diabetes programs should include providing critical equipment such as glucometers to clients to improve the success rate of diabetes social support programs. Ultimately, a greater understanding of these interactions through longitudinal studies is required to investigate all influencers of glycaemic control amongst clients living with Type II diabetes and identify solutions to optimize glycaemic control.

### What is known about this topic

- *Social support is associated with glycemic control among diabetic clients globally;*
- *Informal supporters such as primary caregivers, who include spouses or partners of people living with diabetes (SPWD) who often live in the same household with diabetic patients, are better positioned to provide social support to influence a patient's day-to-day diabetes self-management practices*.

### What this study adds

- *Evidence on the extent of tangible, emotional, and informational social support that clients living with diabetes in Machakos County are receiving;*
- *Policymakers such as the Ministries of Health could employ these findings in designing community-based diabetic care and management programs that wish to leverage primary caregivers in improving chronic disease outcomes;*
- *Evidence on how different types of social support influence glycemic levels among clients living with Type II diabetes in Machakos County, a rural County in Kenya*.
